# β‑Cyclodextrin-Coated *N*‑Doped Carbon Dots for Red-Light-Controlled Nitric Oxide Release and Photothermal Therapy

**DOI:** 10.1021/acsanm.6c02329

**Published:** 2026-07-08

**Authors:** Francesca Laneri, Cristina Parisi, Vittoria Andrigo, Gian Marco Leone, Katia Mangano, Ferdinando Nicoletti, Marta M. Natile, Salvatore Sortino

**Affiliations:** † PhotoChemLab, Department of Drug and Health Sciences, 9298 University of Catania, Catania I-95125, Italy; ‡ ICMATE-CNR Institute of Condensed Matter Chemistry and Technologies for Energy, National Research Council, Department of Chemical Science, University of Padova, Padova I-35131, Italy; § Department of Biomedical and Biotechnological Sciences, University of Catania, Catania I-95125, Italy

**Keywords:** red light, carbon dots, nitric oxide, photothermia, bimodal phototherapy

## Abstract

Photochemically controlled release of nitric oxide (NO) and photothermia are two of the most intriguing unconventional therapeutic approaches to tackle important diseases, including cancer. The development of precursors and strategies to deliver NO and induce photothermal action by exciting suitable precursors in the “therapeutic spectral window” (650–1350 nm) with the tissue-penetrating red light is highly demanding. In this contribution, *N*-doped carbon dots (**NCD**) with absorption extending up to the red region have been synthesized and covalently functionalized with β-cyclodextrins (**βCyD**) at their periphery. The resulting **NCD-βCyD** nanoscaffolds (*ca.* 3 nm in diameter) are dispersible in water medium and able to host a hydrophobic and otherwise blue light-activatable NO photodonor (**1**) within the βCyD cavity. The resulting supramolecular **NCD-βCyD@1** complex is stable in a protein medium and its red light excitation simultaneously leads to (i) satisfactory fluorescence emission, (ii) NO release from **1** via a photoreductive pathway, with an upgrading of about 260 nm in the excitation wavelength, compared to **1** and (iii) efficient photothermal conversion. Due to its emissive properties, the nanoconstruct can be tracked in Caco-2 colon cancer cells, where it localizes mostly at the cytoplasmatic level. Preliminary toxicity experiments carried out with the individual components show that **NCD-βCyD@1** exhibits good biocompatibility in the dark and an enhanced level of cell mortality under the exclusive control of red light against Caco-2 cell lines, due to the combined photodynamic effect of NO released from the guest precursor and the photothermal action of the **NCD-βCyD** core.

## Graphical abstract

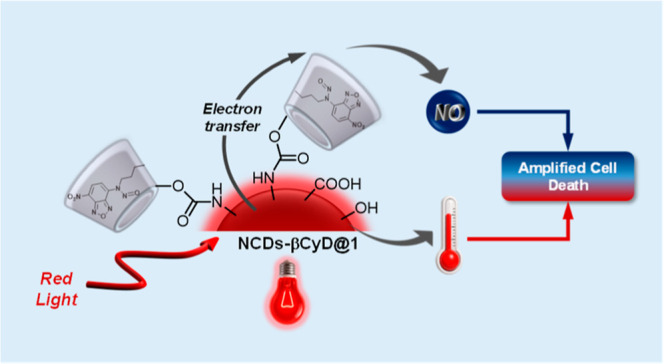

## 1 Introduction

Combination chemotherapy aims to attack tumors from different angles by targeting a single oncogenic pathway through distinct mechanisms or across parallel pathways, without amplifying side effects.^1^ However, due to the high mutation propensity of several cancer types, it remains unclear if chemo-combination will be able to overcome genetic mutation and multidrug resistance (MDR) mechanisms.^2,3^ This has brought multimodal cancer therapy, which combines various therapeutic strategies to target the bulk of tumor cells and resistant cancer cells, to the forefront of cancer research.^4^ Nanotechnology has effectively catalyzed the achievement of multimodal systems, providing a variety of potential solutions to addressing tumor heterogeneity and MDR issues.^5^ Light-controlled generation of “unconventional” therapeutics holds great potential in this regard.^6^ In fact, highly localized cytotoxic bursts can be generated with exquisite spatiotemporal control using suitable photoprecursors by tuning position and intensity of the incident light source. Photodynamic therapy (PDT)^7^ and photothermal therapy (PTT)^8^ are among the best-known unconventional phototherapeutic modalities for cancer and other malignant diseases, with PDT already being used in patients. These treatments exploit photosensitizers (PSs)^7^ and photothermal agents^8^ for the photocatalytic generation of singlet oxygen (^1^O_2_) and heat (Δ), respectively, as the main cytotoxic species. Besides, nitric oxide (NO)-based PDT (NO-PDT), has also come to the limelight, particularly during the past two decades, opening additional and intriguing unconventional phototherapeutic scenarios.^9^ In fact, besides being a key gaseous regulator of many vital functions,^10,11^ NO plays a multiple role in cancer,^12–14^ with dichotomic effects depending on its dose.^15^ This inorganic free radical can act directly as a cytotoxic bullet^12–14^ but also indirectly as an inhibitor of the ABC transporters (efflux pumps) mainly responsible for MDR phenomena,^16–18^ and as an enhancer of local blood flow in hypoxic regions of solid tumors, increasing susceptibility to radiotherapy,^19^ and sensitizing radiation-induced cell death.^20^ In contrast to the working principles of both PDT and PTT, photogeneration of NO is not a catalytic process. It is achieved by NO precursors, namely NO photodonors (NOPs), which covalently incorporate NO into their molecular structure and release it upon homolytic bond cleavage under light excitation.^21–25^ Therefore, the reservoir of NO is univocally dictated by the concentration of the NOP. On the other hand, analogously to ^1^O_2_ and Δ, NO is short-lived (half-life *ca*. 5 s in tissues),^1,2^ thus confining its diffusion radius to the restricted area where it is generated, does not suffer MDR and reacts with all biological targets. Finally, in contrast to ^1^O_2_ and similar to PTT, NO photorelease does not depend on oxygen, making NO-PDT a very powerful approach for treating hypoxic tumors, where PDT usually fails due to low oxygen levels.

Representative examples of different combinations between PDT, PTT and NO-PDT achieved by molecular and macromolecular assemblies and nanomaterials have been illustrated in recent review papers.^26,27^ One of the most demanding requirements for these systems is their activation within the “therapeutic spectral window” (650–1300 nm)^28^ with one photon red or near-infrared (NIR) light excitation. This excitation interval is highly tissue-penetrating due to the high optical transmission of hemoglobin and water.^28^ Additionally, single-photon excitation using conventional light sources is preferable to two-photon excitation with femtosecond pulsed lasers, owing to the high cost, complexity, and limited practicality of the latter.^29^

Carbon dots (CDs) are spherical carbon nanoparticles that can be easily prepared from readily available carbon precursors. They have typical sizes below 10 nm, a core rich in sp^2^-hybridized carbons and a shell bearing different functional groups, i.e., –OH, –COOH, –NH_2_, depending on the synthetic protocols used, which permit surface modification with additional components.^30–32^ Besides their high biocompatibility and cell permeability,^33^ CDs exhibit excellent spectroscopic, photophysical, and photochemical properties, making them intriguing nanoplatforms for applications in photonanomedicine.^34–37^ CDs can exhibit absorption features ranging from the UV to the red/NIR region, as well as emission, which is strongly dependent on the excitation wavelength and useful for biotracking.^38,39^ Besides, ad hoc-prepared CDs can generate ^1^O_2_ and photothermia thus representing a valid alternative to the typical porphyrinoid-based PSs used in PDT and to gold-based nanostructured systems commonly employed in PTT.^40–43^ Finally, CDs can also act as both electron/energy donors and acceptors upon light excitation with suitable chromophoric counterparts.^44,45^

In the frame of phototriggered multimodality, we have recently reported *N*-doped CDs (**NCDs**) covalently integrating an NOP activatable by blue light, demonstrating that light excitation with this wavelength enhances the NO release efficiency through an intramolecular photoinduced electron transfer from the **NCDs** core to the peripheral NOP and, in addition, produces relevant photothermia.^46^ Thereafter, we have also reported that green light excitation of **NCDs** absorbing in this more biocompatible spectral region can trigger NO photorelease from the same NOP covalently integrated at the **NCDs**’surface through a similar photoreductive pathway, with an upgrading of more than 100 nm in the wavelength, and, at the same time, photogenerate ^1^O_2_.^47^

On these grounds, the fabrication of bimodal phototherapeutic nanoconstructs based on **NCDs**, able to generate NO and photothermia simultaneously upon excitation with highly tissue-penetrating red light, is very challenging and represents a significant step forward in the perspective of in vivo studies. To our knowledge, only rare examples are known to date.^48^

In recent years, functionalization of the CDs’surface with cyclodextrins (CyDs),^50,51^ cyclic oligosaccharides formed by six (αCyD), seven (βCyD), and eight (γCyD) glucopyranose units well-known for their capability to encapsulate a variety of molecules with different size and polarity via supramolecular host/guest interactions,^49^ has been demonstrated to be a viable strategy to achieve intriguing biocompatible nanoplatforms for delivery and bioimaging.^50,51^

Motivated by this scenario, we report herein the design, synthesis, characterization, photochemical performances and preliminary biological evaluation of the nanoplatform **NCDs-βCyD@1** (Scheme 1). It consists of **NCDs** covalently integrating βCyD units, which are able to encapsulate the otherwise blue light activatable NOP **1**. We show that this nanoconstruct is stable in a protein medium, internalizes in cancer cells, where it can be tracked due to its unambiguous red emission, and that the selective excitation of the **NCDs** core with the highly biocompatible red light induces NO release from the NOP **1**, more likely by photoinduced electron transfer, and remarkable photothermal effect, resulting in enhanced anticancer efficacy by bimodal cell killing action.

**Scheme 1:**
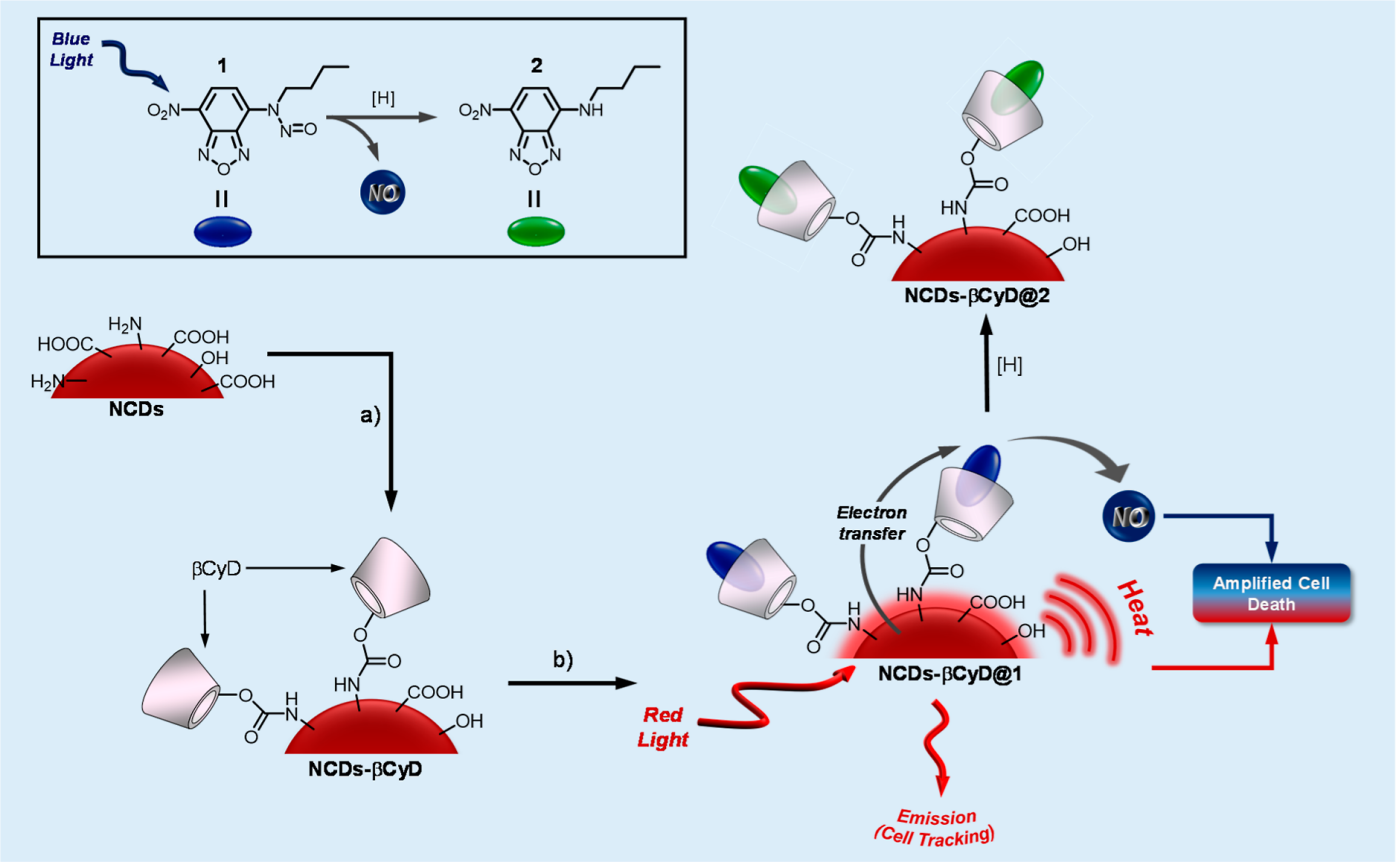
Preparation of **NCDs-βCyD**, the Supramolecular Nanoplatform **NCDs-βCyD@1** and its Working Principle. (a) NCDs, βCyD, PBS, 4 days, 50 °C. (b) **1**, PBS, 3h, r.t. The Inset Shows the Molecular Structures of the NOP **1** and its Stable Photoproduct **2** Formed after NO Release Induced by Blue Light. At pH 7.4, the Carboxylic and the Amino Groups of **NCDs** are Expected to be Predominantly Present under their Negative and Positive Ionized Forms, Respectively

## 2 Experimental Section

### 2.1 Materials and Methods

All chemicals were purchased by Sigma-Aldrich and used as received. All solvents used (Sigma-Aldrich) were spectrophotometric grade. Deionized ultrafiltered water was used throughout this study.

### 2.2 Synthesis

The synthesis of the **NCDs** was carried out according to a previously reported protocol, with some modifications.^52^ Citric acid (2 g) and urea (4 g) were dissolved in 20 mL of DMF, followed by the addition of NH_4_F (0.195 g). The mixture was sonicated for 10 min and then transferred into a Teflon-lined stainless-steel autoclave, where it was heated at 180 °C for 4 h. After cooling to room temperature, the resulting red suspension was treated with NaOH aqueous solution (pH = 13), stirred for 10 min, and centrifuged (12,000 r min-1, 10 min). The black precipitate obtained was washed repeatedly with dilute HCl (pH = 2) and ultrapure water, then collected by centrifugation and freeze-dried for 48 h.

A previously reported protocol inspired the synthesis of the **NCDs-βCyD**.^53^ The amine groups on the surface of **NCDs** were exploited to conjugate with the hydroxyl groups of βCyD monomers through a 1,1′-Carbonyldiimidazole (CDI)-mediated coupling reaction. To this end, **NCDs** (500 mg) were dissolved in 5 mL of PBS (pH 7.4, 10 mM) and dropwise added to a solution of βCyD (3.6 g, 3.2 mmol) and CDI (516.6 mg, 3.2 mmol) in PBS (5 mL). The mixture was stirred at 50 °C for 4 days, then filtered and dialyzed against deionized water (MWCO = 2 kDa) for 48 h. The resulting **NCDs-βCyD** conjugate was then lyophilized and stored at room temperature.

NOP **1** was synthesized according to our reported procedure.^54^

### 2.3 Preparation of the Supramolecular Complex

**NCDs-βCyD@1. NCDs-βCyD** and, for comparison, **NCDs** were first dissolved in Milli-Q water and sonicated for 15 min, followed by filtration. They were then diluted to the desired concentration, and PBS (pH 7.4, 10 mM) was added to achieve the appropriate buffer conditions. NOP **1** powder was subsequently added, and the mixtures were stirred in the dark for approximately 3 h. Finally, the solutions were filtered prior to use.

### 2.4 Fluorescence Quantum Yield

Fluorescence quantum yields (Φ_f_) were determined in PBS solutions at λ_exc_ = 560 nm using optically matched dispersions at the excitation wavelength of the different samples and Methylene Blue (Φ_f_ = 0.04) as a standard^55^ through eq 1

1 $$\Phi_{f}=\frac{\Phi_{f\left(s\right)}I}{I_{\left(s\right)}}$$

where Φ_f(s)_ is the fluorescence quantum yield of the standard; I and I_(s)_ are the areas of the fluorescence spectra of either **NCDs-βCyD** or **NCDs-βCyD@1**, and the standard, respectively. In all cases, absorbance at the excitation wavelength was less than 0.1.

### 2.5 Photothermal Conversion Efficiency

Photothermal conversion efficiency η was calculated according to the literature,^56,57^ using the following eq 2

2 $$\eta =\frac{hS\left(T_{\max}-T_{surr}\right)-Q_{dis}}{I\left(1-10^{-A}\right)}$$

where *h* represents the heat transfer coefficient, and *S* denotes the surface area of the container.

### 2.6 Cell Lines and Culture Conditions

The human colorectal cancer cell line Caco-2 (HTB-37) was obtained from the American Type Culture Collection (ATCC, Manassas, VA, USA). Cells were cultured in Dulbecco’s Modified Eagle Medium (DMEM; Sigma-Aldrich, St. Louis, MO, USA) supplemented with 2 mmol/L l-glutamine, 100 IU/mL penicillin, 100 μg/mL streptomycin, and 20% heat-inactivated fetal bovine serum (FBS; Sigma-Aldrich). Cultures were maintained at 37 °C in a humidified atmosphere containing 5% CO_2_. The absence of mycoplasma contamination was verified by PCR assay. All experiments were performed using cells within 15 passages after thawing.

### 2.7 Fluorescence Microscopy Imaging

For fluorescence analyses, Caco-2 cells were seeded at a density of 2.0 × 10^5^ cells/mL in poly l-lysine-coated μ-Slide 8-well chambers (Ibidi, Germany). Following a 24 h incubation at 37 °C and 5% CO_2_, the cells were treated with **NCDs-βCyD**, **NCDs-βCyD@1** or DMSO (control) for 2 h. After washing, cellular nuclei and mitochondria were stained with NucBlue Dye (R37605, Thermo Fisher Scientific, Waltham, MA, USA) and MitoTracker Green FM Dye (M46750, Thermo Fisher Scientific, Waltham, MA, USA), respectively, according to the manufacturer’s instructions.

High-resolution images were subsequently acquired on a Leica TCS SP8 confocal microscope. Excitation was performed using 405, 488, and 561 nm laser lines, and fluorescence emission was collected within the ranges of 420–480 nm (blue channel), 500–520 nm (green channel), and 610–670 nm (red channel).

### 2.8 Cell Viability Assay

Caco-2 cells were seeded into 96-well culture plates (Nunc, Denmark) at a density of 2 × 10^5^ cells/mL and allowed to adhere for 24 h under standard culture conditions. After attachment, cells were treated with **1** (incubated in a DMSO solution), **NCDs-βCyD**, or **NCDs-βCyD@1** for 2 h. Control samples were treated with the corresponding vehicle (DMSO) at an equivalent final concentration. At the end of the treatment period, cells were carefully washed with phosphate-buffered saline (PBS) to remove residual compounds and were subsequently maintained in the dark at 37 °C or irradiated at the same temperature with red light at 671 nm (200 mW cm^–2^) for 30 min using a beam expander. Immediately after irradiation, fresh complete culture medium was added to each well, and cells were incubated for an additional 48 h to allow the development of treatment-induced effects before subsequent analyses.

The 3-(4,5-Dimethylthiazol-2-yl)-2,5-diphenyl tetrazolium bromide (MTT, Sigma-Aldrich, St. Louis, MO, USA) assay was used to assess cellular viability. Briefly, after the indicated time, MTT solution was added to each well at a final concentration of 0.5 μg/mL, followed by incubation for 4 h to allow formazan crystal formation. The insoluble crystals were solubilized using acid-isopropanol stop solution (0.04 N HCl), and absorbance was measured at 492 nm using a BioTek 800 TS microplate reader (Agilent Technologies, Santa Clara, CA, USA). Cell viability was expressed as a percentage relative to untreated control cells, assumed to be 100% viable.

All experiments were performed in three independent biological replicates, each technical replicate conducted in triplicate. Data are presented as mean ± standard deviation (SD).

### 2.9 Statistical Analysis

Data manipulation and analysis were performed using GraphPad Prism version 9.0 for Windows (GraphPad Software, San Diego, CA, USA). Data normality was evaluated using the Shapiro–Wilk test, confirming a normal distribution of the samples and supporting the use of parametric statistical analyses. Comparisons between two groups were performed using a two-tailed unpaired Student’s *t*-test, whereas differences among three or more groups were assessed by one-way analysis of variance (ANOVA) followed by Tukey’s post hoc multiple comparisons test. Statistical significance was defined as a *p*-value <0.05. Levels of significance were indicated as follows: *p* < 0.05 (*), *p* < 0.001 (**), and *p* < 0.0001 (***, ### or ●●●).

### 2.10 Instrumentations

High-resolution transmission electron microscopy (HR-TEM) analysis and X-ray photoelectron spectroscopy (XPS) measurements were performed with the instrumentation and procedures already described.^47^

FTIR spectra were recorded with a System 2000 (PerkinElmer, Waltham, MA, USA).

Thermogravimetric analysis (TGA) was performed using a PerkinElmer TGA 4000 instrument under a prepurified nitrogen atmosphere at 1 atm. Measurements were carried out at a heating rate of 10 °C/min over a temperature range of 50–800 °C.

Apparatus for steady-state UV–vis absorption and emission as well as time-resolved emission were already reported.^47^ For fluorescence lifetime measurements, the samples were irradiated by a pulsed diode excitation source Nanoled at 560 nm and the kinetics were monitored at 630 nm. The exponential fit of the fluorescence decay was obtained using eq 3

3 $$I\left(t\right)=\Sigma \alpha_{i}\exp\left(-t/\tau i\right)$$

Steady-state irradiation experiments were performed in a thermostated quartz cell (1 cm path length, 3 mL capacity) under gentle stirring using a CW red laser (ca. 200 mW cm^–2^) at λ = 671 nm having a beam diameter of ca. 1.5 mm. The photolysis experiments under anaerobic conditions were performed by deoxygenating the dispersion via vigorous, constant bubbling with pure nitrogen (previously saturated with solvent).

Photothermal experiments were performed as already described in our previous paper.^46^

Direct monitoring of NO release was performed using an amperometric World Precision Instruments ISO–NO meter by using our already reported procedures.^46,47^

## 3 Results and Discussion

### 3.1 Design, Synthesis and Structural Characterization

This work was inspired by our recent findings demonstrating that NO release from the NOP **1** (see inset in Scheme 1), otherwise activatable by the poorly tissue-penetrating blue light,^54^ can be triggered with the much more biocompatible red light by photoexcitation of different porphyrinoid-based photosensitizers (PSs), used as red light harvesting antennae.^58,59^ The NO photorelease takes place through a photoreductive pathway mediated by an intermolecular photoinduced electron transfer occurring in a photocatalytic fashion between the PSs and **1** supramolecularly coencapsulated within several types of biocompatible nanocarriers.^58,59^ Thereafter, this general strategy inspired other groups to trigger NO release from different nitroso-derivatives co-encapsulated with metal based PSs within different nanocarriers.^60^ The fast NO detachment is encouraged by the formation of the **1** ^**.‑**^ radical anion which, analogously to other nitroso-derivatives, has a lower dissociation energy of the *N*–NO bond than the neutral form.^61^

On these grounds, and encouraged by our recently discovered capability of blue and green light absorption **NCDs** to trigger NO release from nitroso-derivatives linked at their surface upon excitation in this wavelength range,^46,47^ we devised the nanoplatform **NCDs-βCyD** (see Scheme 1). It consists in **NCDs** characterized by a spectral absorption extending in the red region, covalently decorated with multiple βCyD units at the surface. The final goal was to exploit the βCyD cavities of **NCDs-βCyD** to host the highly hydrophobic NOP **1**, leading to the supramolecular construct **NCDs-βCyD@1**, in which the **NCDs** core is expected to play a multiple role as (i) electron donors to trigger NO photorelease from the guest, (ii) PTT agent to induce photothermia and (iii) emissive component to generate fluorescence useful for cell tracking.

**NCDs** were synthesized with slight modifications of a previously reported synthetic protocol and further covalently functionalized with βCyD via a carbamate linkage (see experimental and Scheme 1). HR-TEM micrographs of pristine **NCDs** show quite dispersed, spherical-like shaped nanocrystals with a mean diameter of 2.9 ± 0.8 nm (Figure 1A).

**Figure 1:**
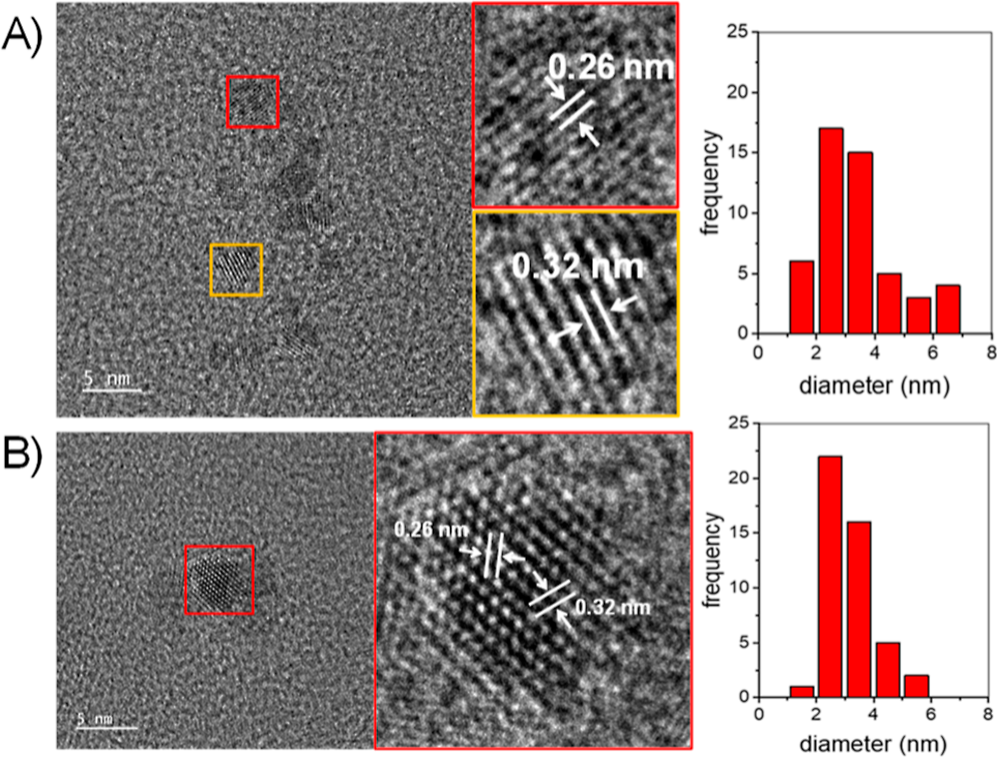
HR-TEM micrographs and size distribution of (A) **NCDs** and (B) **NCDs-βCyD**.

They reveal clear lattice fringes, with d-spacing of 0.26 and 0.32 nm corresponding to the (020) and (002) planes, respectively. These findings are consistent with previously synthesized **NCDs** ^46^ and graphitic carbon.^62,63^ No significative changes are observed after conjugation with βCyD: both **NCDs** mean diameter (3.0 ± 0.5 nm) and crystallinity are preserved in the case of **NCDs-βCyD** (Figure 1B). The XPS analysis was performed to evaluate the composition as well as the chemical state and environment of the synthesized material. The pristine **NCDs** (Figure 2) reveal the presence of C (72.0 at %), N (5.3 at %), O (22.7 at %) and traces of Na. The analysis of C 1s, N 1s, and O 1s core levels confirms the formation of **NCDs**.^47^ Specifically, the deconvolution of the C 1s reveals the presence of four distinct carbon species centered at 284.8 eV (C=C, C–C), 285.8 eV (C–O, C–N), 287.1 eV (C=O, C=N), and 288.8 eV (O=C–OH), respectively. The N 1s region can be fitted with four components, showing maximum intensities at 398.4, 399.4, 400.1, and 401.5 eV, consistent with pyridinic-N, aminic R–NH_2_, pyrrolic-N, and graphitic-N, respectively. Finally, the O 1s region consists of three contributions at 530.8, 531.8, and 533.2 eV, ascribable to C–OH, C=O, and O–C–O. The small contribution at 534.5 eV is ascribable to KLL of Na. The binding energies and atomic percentages of the different species are summarized in Table S1.

**Figure 2:**
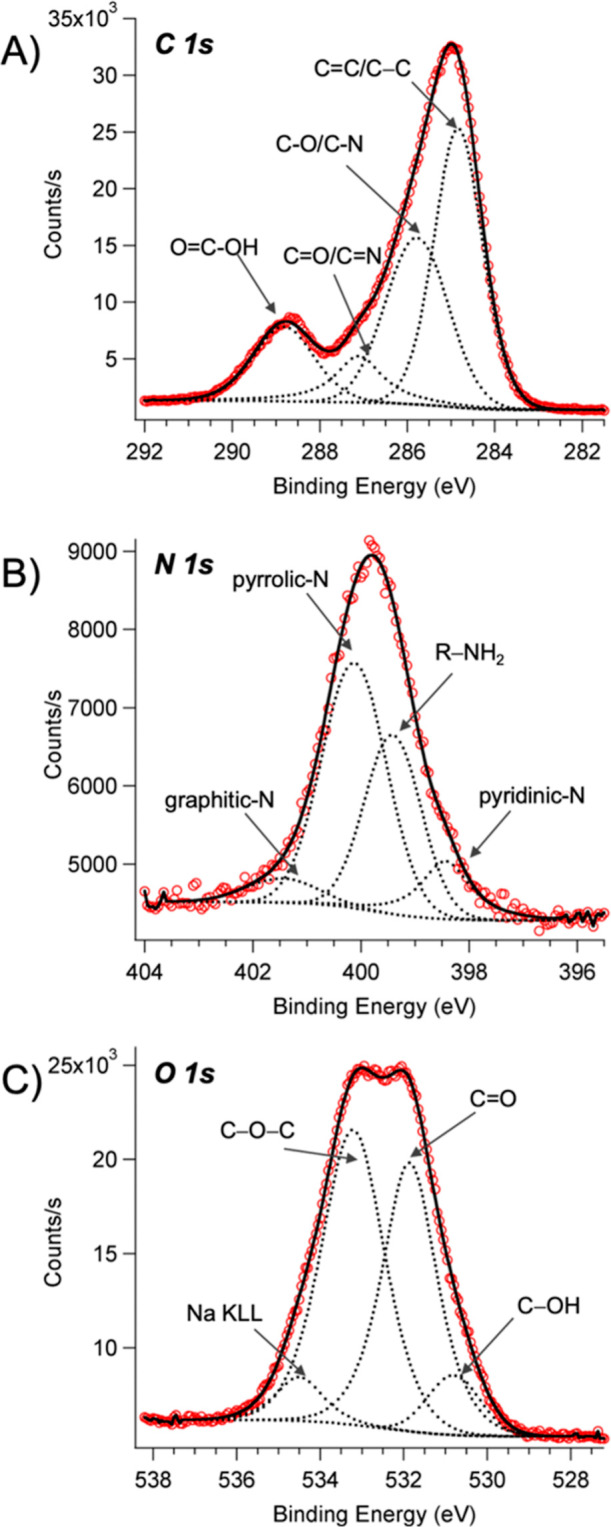
XPS analysis of **NCDs**: high resolution spectra of (A) C 1s, (B) N 1s, and (C) O 1s. Red circles for raw data; black continuum lines and dashed lines for the fitting curves.

The XPS analysis of **NCDs-βCyD** reveals several important changes (Figure S1 and Table S2). A change in relative amount of C (66.6 at %), N (0.5 at %) and O (32.9 at %) is observed. This is consistent with the presence of a βCyD coating on the NCDs surface. Moreover, as expected, the fitting of the C 1s and O 1s core levels reveals multiple contributions with slightly different binding energies compared to those observed for pristine **NCDs**. These differences, indicative of a modified chemical environment, are primarily attributed to the carbon and oxygen species of the βCyD units present on the **NCDs** surface.

FTIR analysis of **NCDs** (**a** in Figure 3A) show a broad signal from 3660 to 2150 cm^–1^, arising from surface OH/NH groups, which ensure good dispersibility in water and suitability for further functionalization, and peaks at 1570 cm^–1^, 1345 cm^–1^, and 1196 cm^–1^ corresponding to C=C stretching, semi-ionic C–F, and covalent C–F stretching, respectively.^47^ The successful formation of the carbamate linkage between the βCyD and the **NCDs** is suggested by the appearance of a well-defined peak at 1653 cm^–1^ (**b** in Figure 3A).^53^ This signal is shifted with respect to the H–O–H bending vibration of βCyD (**c** in Figure 3A) and is clearly different from the broader COO- signal of bare **NCDs**, which originates at 1792 cm-.^1^ For sake of comparison, we also carried out a control experiment with a physical mixture of **NCDs** and βCyD prepared without the coupling agent CDI. The related FTIR spectrum of this sample (Figure S2) shows a broader and less defined band in this region, suggesting that the observed spectral feature is not merely due to the coexistence of the two components, being consistent with successful functionalization. Further evidence for the successful grafting of βCyD onto the **NCDs** surface is provided by the spectral features appearing in the 1000–1150 cm^–1^ region. Specifically, the **NCDs-βCyD** spectrum exhibits sharp peaks at 1155 and 1036 cm^–1^, closely matching the characteristic pattern observed for pristine βCyD, and attributable to glycosidic C–O–C stretching and C–O vibrations of the glucopyranose units, respectively.

**Figure 3:**
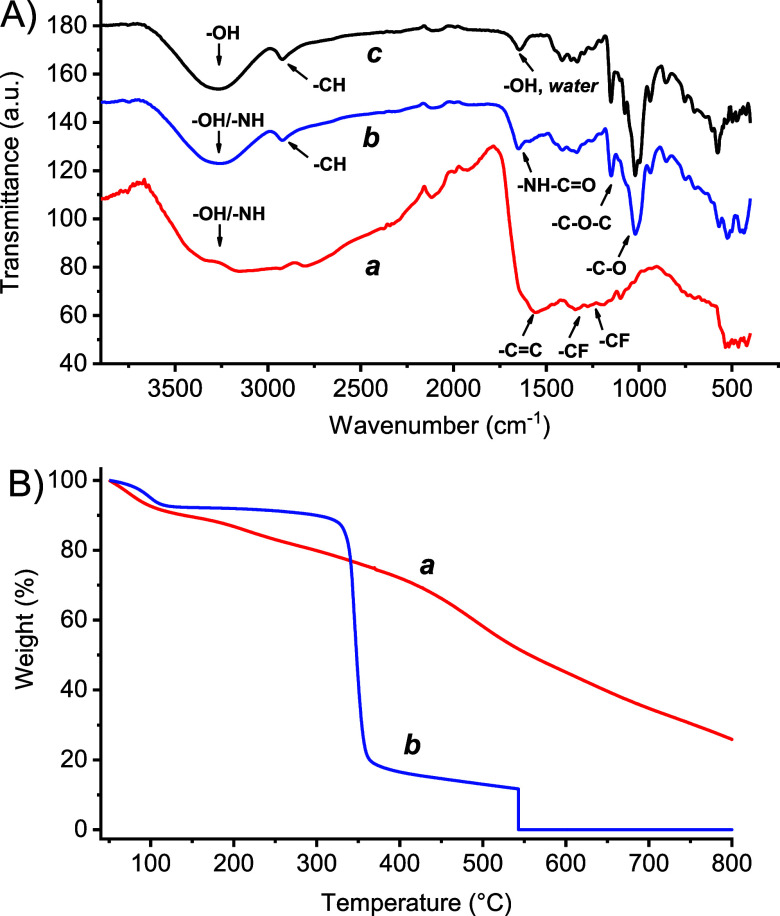
(A) FTIR spectra of **NCDs** (**a**), **NCDs-βCyD** (**b**) and βCyD (**c**). (B) TGA analysis of **NCDs** (**a**) and **NCDs-βCyD** (**b**).

The presence of successful linkage of βCyD on the surface of **NCDs** was also demonstrated by thermogravimetric analysis (TGA). Pristine **NCDs** exhibited the onset of mass loss at approximately 150 °C (**a** in Figure 3B), which can be attributed to the thermal decomposition of oxygen-containing surface functional groups.^64^ The total residue reached ca. 37.47 wt % at 600 °C. In contrast, the **NCDs-βCyD** exhibited an initial mass

loss at around 110 °C, attributed to the loss of adsorbed moisture, followed by a sharp mass loss starting at approximately 350 °C, corresponding to the thermal degradation of βCyD, which occurs after the melting of the glucose units within the cyclodextrin structure^65^ (**b** in Figure 3B). At 600 °C, the overall residue amounted to approximately 79.72 wt %. Based on the difference between the two profiles, the **βCyD** content grafted onto the CDs was estimated to be about 42.25 wt %.^64^ Notably, the abrupt mass-loss step observed at ca. 600 °C for **NCDs-βCyD** is attributable to the decomposition of the βCyd moieties covalently bound to the NCDs surface. The significant shift of the decomposition temperature compared to free **βCyD**, typically observed at 300–400 °C, suggests enhanced thermal stability arising from interfacial interactions with the **NCDs** and the possible formation of a more compact hybrid organic-carbonaceous domain. Furthermore, comparison with a physical mixture of **NCDs** and βCyD revealed a markedly different degradation profile (Figure S3).

### 3.2 Spectroscopic and Photochemical Behavior

**NCDs-βCyD** were well dispersible in PBS medium and exhibited an UV–Vis absorption spectrum characterized by a broad absorption band spanning the whole Vis region, with a maximum at ca. 540 nm and extending into the NIR (**a** in Figure 4A). This absorption is generally ascribed to π → π* transitions of extended sp^2^-conjugated domains and to *n* → π* transitions associated with functional groups containing heteroatoms, typical of **NCDs**.^66–68^ These spectral features basically overlap to those of naked **NCDs** (**b** in Figure 4A), suggesting that, according to literature, the covalent linking with βCyD does not perturb the electronic structure of the **NCDs** core.^50,51^

**Figure 4:**
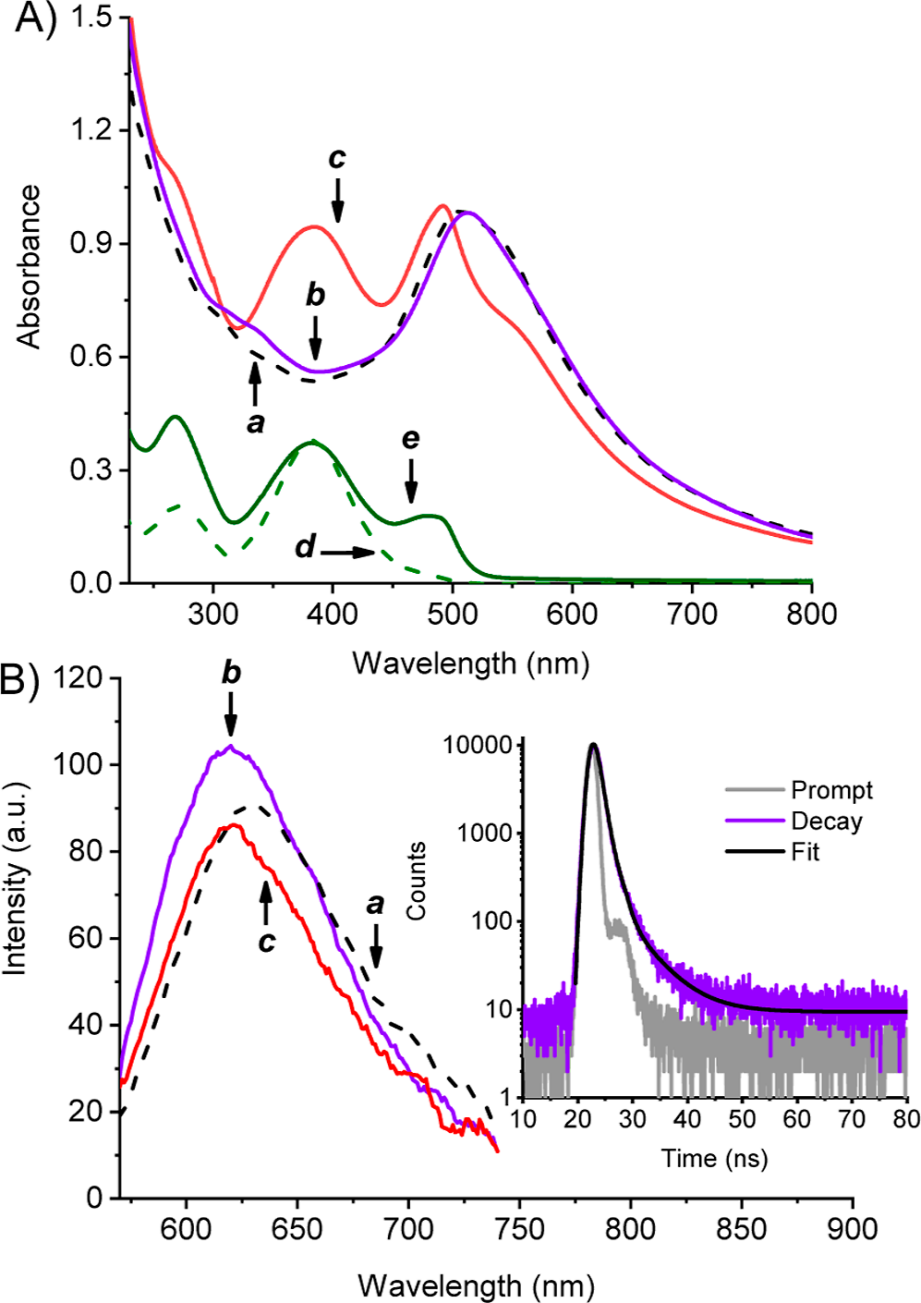
(A) Absorption spectra of aqueous PBS (pH 7.4, 10 mM) dispersions of **NCDs** (**a**), **NCDs-βCyD** (**b**) and **NCDs-βCyD@1** (**c**). Absorption spectra of **1** in methanol (**d**) and in the presence of an aqueous solution of βCD (**e**). (B) Fluorescence emission spectra of aqueous PBS dispersions of **NCDs** (**a**), **NCDs-βCyD** (**b**) and **NCDs-βCyD@1** (**c**) at λ_exc_ = 560 nm. The inset shows the fluorescence decay, λ_exc_ = 560 nm and λ_em_ = 630 nm, and the related biexponential fitting of **NCDs-βCyD**. [**NCDs**] (0.7 mg mL^–1^); [**NCDs-βCyD**] = 12.6 mg mL^–1^); [**1**] = 25 μM; [βCyD] = 10 mM. *T* = 25 °C.

NOP **1** is not soluble in water medium, however, it resulted soluble in the presence of aqueous dispersions of **NCDs-βCyD** as evidenced by the appearance of its typical absorption of **1** characterized by a main maximum at *ca*. 380 nm and a new absorption at *ca*. 480 nm (***c*** in Figure 4A). This latter was much more pronounced than that observed in methanol, where **1** solubilizes well (**d** in Figure 4A), but very similar to that observed for **1** in the presence of aqueous solution of isolated βCyD (**e** in Figure 4A). These results are in line with the probable encapsulation of **1** within the hydrophobic βCyD cavities to form the **NCDs-βCyD@1** supramolecular complex (vide infra). Note that, the absorption band of the **NCDs-βCyD** at 540 nm is strongly modified and accompanied by the formation of a shoulder at ca. 560 nm in the case of the **NCDs-βCyD@1** complex. These spectral changes cannot be ascribed to a mere arithmetic contribution of **1** since it does not absorb beyond 530 nm. Rather, based on the strong electron-acceptor nature of **1** and the electron-donor property of **NCDs**, these changes can result from a ground-state electronic interaction between the **NCDs** core and the encapsulated **1**.

**NCDs** show emission strongly dependent on the excitation wavelength, a typical phenomenon for this type of nanostructures,^38,39^ covering the entire Vis range (Figure S4). In the perspective of exploiting these emission properties for cell tracking, we specifically explored the emissive behavior at λ_exc_ = 560 nm. This excitation wavelength induces red emission of **NCDs** with λ_max_ = 630 nm (**a** in Figure 4B), which is beyond the ambiguous typical range of cells autofluorescence, with quantum yield Φ_f_ = (1.5 ± 0.1)×10^–2^. **NCDs-βCyD** exhibits a 10 nm of blue shift of the emission maximum (**b** in Figure 4B) and an increase of Φ_f_ to (1.9 ± 0.1)×10^–2^. **NCDs-βCyD@1** shows identical spectral shape to the uncomplexed analogue (***c*** in Figure 4B) and a reduction of Φ_f_ to (1.4 ± 0.1)×10^–2^. Such a quenching accounts for an interaction between **1** and the **NCDs** core in the excited state. Time-resolved fluorescence measurements also confirmed this. **NCDs-βCyD** exhibit a biexponential decay, typical for CDs and reflecting different fluorogenic domains,^38,39^ with lifetimes (τ) and related amplitudes (α) being τ_1_ = 1.03 ns, α_1_ = 94.23% and τ_2_ = 5.00 ns, α_2_ = 5.77% (inset Figure 4B), respectively. In the case of **NCDs-βCyD@1** we observed no change in the lifetime of the shorter component τ_1_ but a reduction of its amplitude to α_1_ = 89.27%, and a shortening of the lifetime of the longer component to τ_2_ = 3.75 ns accompanied by an increase of its amplitude to α_2_ = 10.73% (Figure S5).

In this regard, we rule out that the emission quenching observed is due to any energy transfer via FRET mechanism between the **NCDs** core and the peripheral **1**. In fact, the emission of the former (energy donor) falls beyond the absorption of the latter (energy acceptor), resulting in a lack of spectral overlap, an indispensable requisite for FRET, making this process thermodynamically uphill. More likely, as observed in our recent works,^46,47^ a photoinduced electron transfer from **NCDs**, acting as electron donors, to the strong electron acceptor **1** seems the most probable quenching route. This hypothesis accords well with the ground-state interactions discussed above (vide supra), which are probably charge-transfer in nature, in line with what has been reported by Guldi et al. for CDs nanoconjugates functionalized with strong electron-acceptor chromophoric components.^69^ Photolysis experiments were then carried out under red light at λ_exc_ = 671 nm. Under these conditions, **NCDs-βCyD** did not show any significant changes in their absorption spectra, in accordance with excellent photostability (data not shown). In contrast, red light irradiation of the complex **NCDs-βCyD@1** resulted in a bleaching of the absorption band at 380 nm and growth of a new absorption at *ca*. 480 nm (Figure 5A). The photolysis profile is characterized by four well-defined isosbestic points, indicative of a clean photochemical process. This spectral evolution is in excellent agreement with that observed upon direct irradiation of **1** with blue light (Figure S6) and consistent with the NO release accompanied by the formation of the stable photoproduct **2** (see inset Scheme 1).^54^ Since no formation of precipitate was observed during the photolysis, it is reasonable to suggest that, given its hydrophobic features, photoproduct **2** remains more likely encapsulated in the cavity of **NCDs-βCyD** as supramolecular inclusion complex (see Scheme 1).

**Figure 5:**
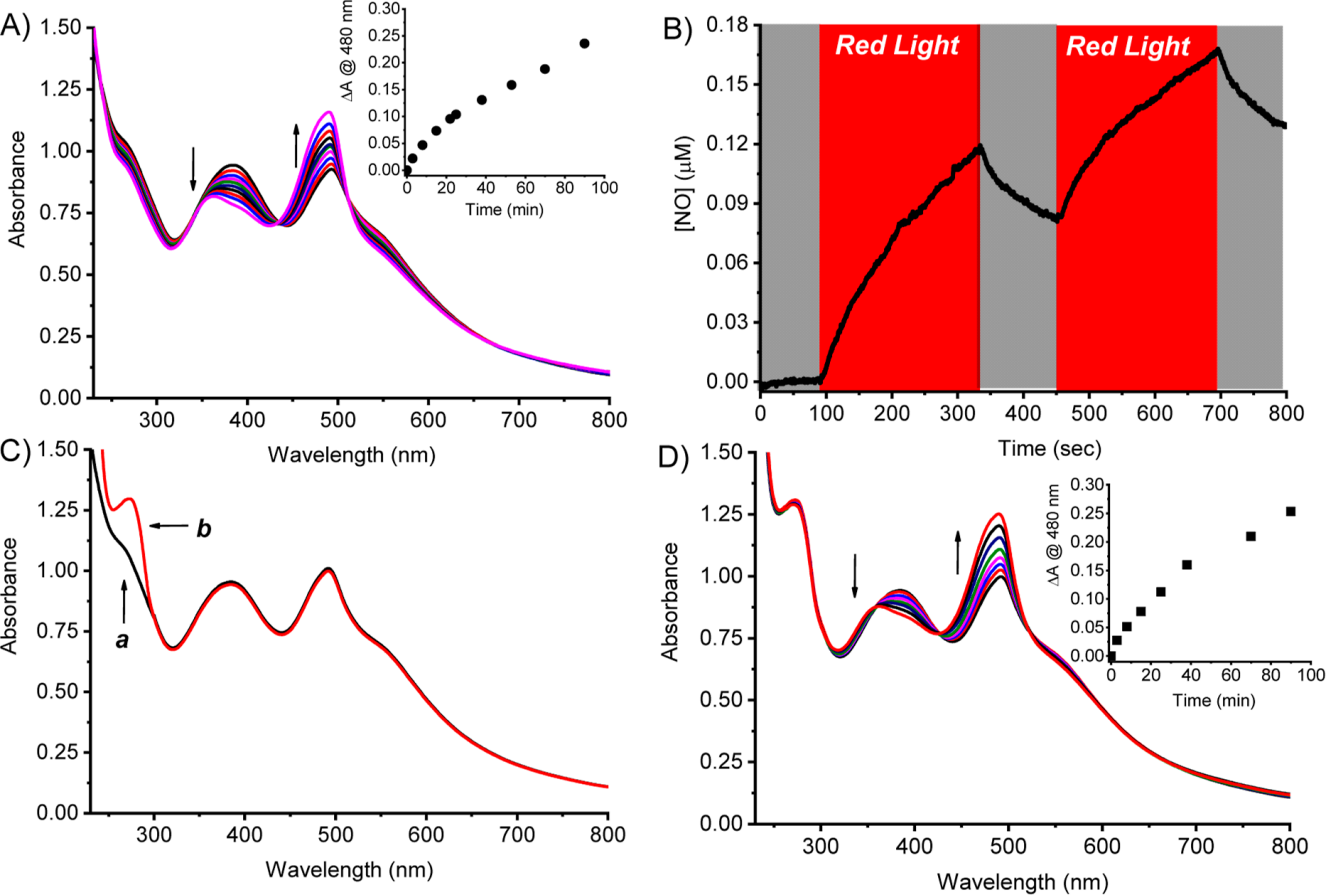
(A) Absorption spectral changes observed upon irradiation of an air-equilibrated PBS (pH 7.4, 10 mM) dispersion of **NCDs-βCyD@1** at λ_exc_ = 671 nm at different times from 0 to 90 min. The arrows indicate the course of the spectral profile with the illumination time. The inset shows the difference of absorbance observed at 480 nm. (B) NO release profile observed for the same sample as in (A) upon alternate cycles of light irradiation at λ_exc_ = 671 nm. (C) Absorption spectra of the same sample as in (A) in the absence (**a**) and after 1h of incubation with BSA at 37 °C (**b**). The absorption band at 274 nm is due to BSA. (D) Absorption spectral changes observed upon irradiation of sample **b** in Figure 5C with λ_exc_ = 671 nm at different times from 0 to 90 min. The arrows indicate the course of the spectral profile with the illumination time. The inset shows the difference of absorbance observed at 480 nm. [**NCDs-βCyD**] = 12.6 mg mL^–1^; [**1**] = 25 μM; [BSA] = 0.8 mg mL^–1^. *T* = 25 °C.

NO release under red light excitation from **NCDs-βCyD@1** was then unambiguously demonstrated by its direct amperometric detection. As shown in Figure 5B, NO release is observed exclusively under red light illumination of the sample whereas immediately stops under dark conditions.

Since the absorption of **1** does not extend beyond 550 nm (see **e** in Figure 4), this red light-triggered NO release cannot be due to the direct absorption of the NOP. Besides, photoinduced energy transfer between the **NCDs** and **1** is definitely ruled out because thermodynamically uphill. Rather, it can be tentatively ascribed to the photoinduced electron transfer discussed before and responsible for the fluorescence quenching, involving the **NCDs** as the electron donor core and peripheral **1** as the electron acceptor. As outlined in the introductory part, such a type of photoredox mechanism has been already proven for other *N*-nitrosoderivatives photosensitized by green light-absorbing **NCDs** ^46^ and red light-absorbing phorphyrinoid-based PSs^58,59^ and encourage the NO detachment by the formation of the radical anion centered on the nitroso group, which has a much low dissociation energy of the *N*–NO bond compared with the neutral form.^61^

Given their biological applications, the stability and photoreactivity of **NCDs-βCyD@1** were also tested in a protein medium under physiological conditions. To this end, the complex was incubated with bovine serum albumin (BSA) at 37 °C in the dark for 1 h and then irradiated with red light. The unaltered absorption spectral profile shown in Figure 5C suggests that, despite its supramolecular character, **NCDs-βCyD@1** remains stable in the biological medium and that no displacement of **1** by BSA takes place. This protective effect likely arises from the hydrophilic and sterically demanding nature of the βCyD corona, which reduces nonspecific protein adsorption and mitigate protein corona formation, preserving the optical and functional properties of the photoactive complex.^70^

Besides, the photolysis profile and related kinetic behavior under these conditions (Figure 5D) were very similar to those obtained in the absence of BSA (see Figure 5A for sake of comparison). Note that, such a result is not trivial. In fact, the photochemical properties of a specific photoactive construct can be significantly influenced by competitive, undesired photoreactions with BSA, which can reduce efficiency or even suppress the main photolytic process.

Comparative experiments carried out with a mixture of naked **NCDs** and **1** revealed the βCyD unit as a key component in preserving both the stability and the photoreactivity of the complex in the biological medium. In fact, the insoluble NOP **1** also became soluble in the presence of aqueous dispersions of **NCDs** (**a** in Figure S7A), probably by a physical adsorption at their surface. However, in contrast to what was observed for **NCDs-βCyD@1**, the addition of BSA led to a modification of the absorption spectral profile (**b** in Figure S7A), according with an interaction of **1** with the protein.

Moreover, in contrast to what was observed for **NCDs-βCyD@1**, when the **NCDs/1** mixture was irradiated with red light under the same experimental conditions, no significant spectral changes were observed (Figure S7B), suggesting complete suppression of **1**’s photochemical reactivity. This finding is in good agreement with a physical adsorption of **1** directly at the **NCDs** surface. It is more likely the result of an effective quenching process due to the very close proximity of the two components, which is competitive with the NO photorelease.

The photothermal conversion capability of **NCDs-βCyD** and its complex with **1** was explored by excitation at 671 nm. Figure 6A shows a significant, comparable temperature increase within a few minutes upon irradiation of both samples. As a comparison, the irradiation of pure PBS did not show any significant temperature change in the solution. A representative collection of thermal images directly shows that the temperature of a suspension of **NCDs-βCyD@1** rises from *ca*. 20 °C to *ca*. 32 °C in less than 10 min upon exposure to a red laser (Figure 6B). Therefore, these results demonstrate that the **NCDs-βCyD@1** nanoplatform, in addition to generating NO, produces distinct photothermal effects. Besides, **NCDs-βCyD@1** exhibited an excellent photothermal stability as proven by the highly reproducible photothermal performances after three cycles of light/dark treatments (Figure 6C). Figure 6D shows the plot of the cooling time versus—ln­(θ) (i.e., the negative natural logarithm of the temperature driving force obtained from the cooling state. The conversion efficiency η (see the Experimental section for detailed calculations) was *ca*. 35%, in agreement with that reported for CDs and other carbon-based photothermal agents.^42,43,48^

**Figure 6:**
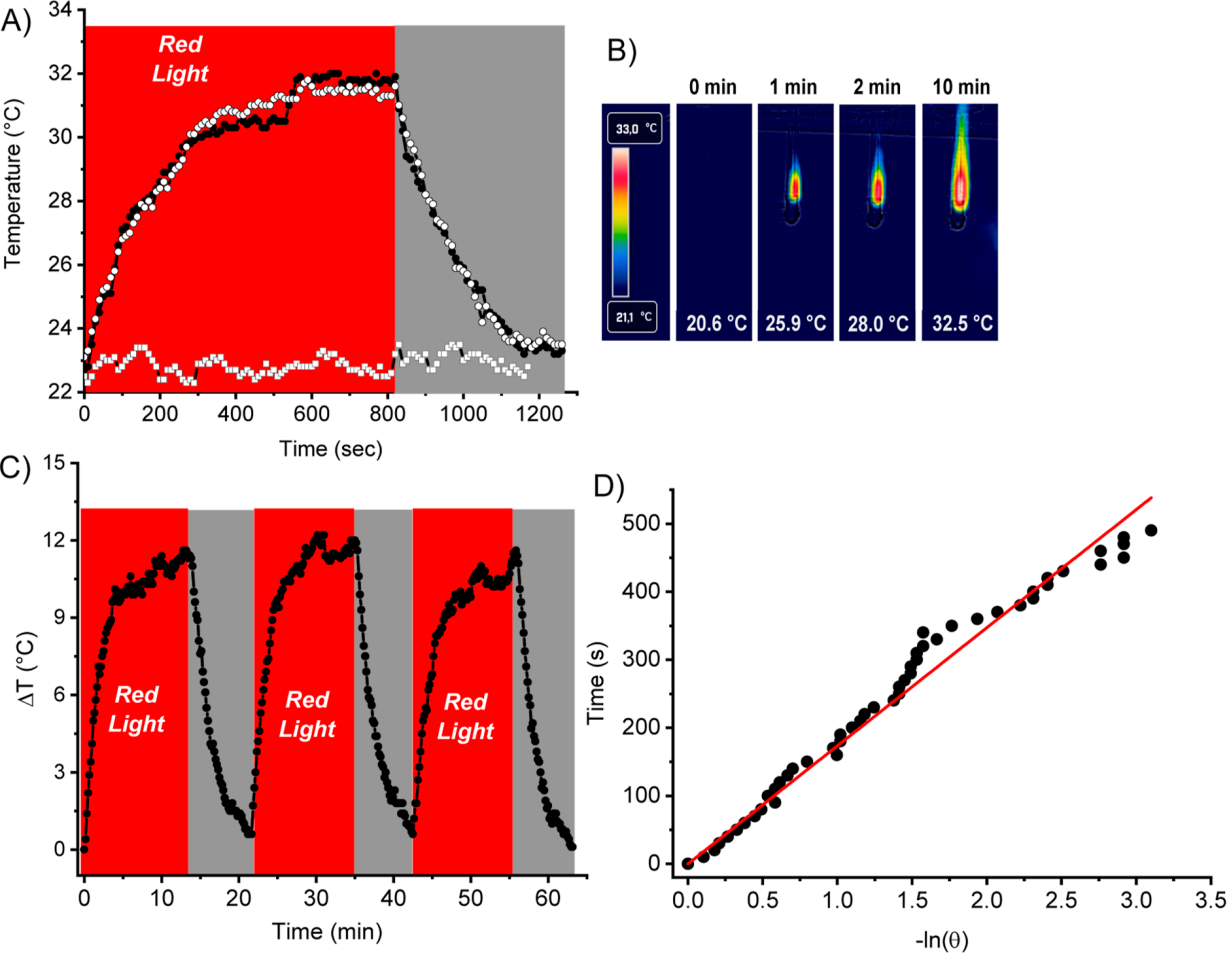
(A) Temperature changes observed upon 671 nm light excitation of air-equilibrated PBS (pH 7.4, 10 mM) dispersion of **NCDs-βCyD** (◯), **NCDs-βCyD@1** (●) and, for comparison, neat PBS (□). (B) Representative thermographic images of the **NCDs-CyD@1** sample recorded at different illumination times. (C) Temperature difference changes observed for the **NCDs-βCyD@1** sample under three ON/OFF irradiation cycles. (D) Linear time data versus—ln­(θ) obtained from the cooling period of the **NCDs-βCyD@1** sample as in (A). [**NCDs**] (0.7 mg mL^–1^); [**NCDs-βCyD**] = 12.6 mg mL^–1^); [**1**] = 25 μM.

In view of this photothermal action and considering that temperature-induced NO release is not uncommon for *N*-nitroso derivatives one could reasonably argue that the NO release observed under red light excitation of **NCDs-βCyD@1** (Figure 5A,B) is not due to the proposed photochemical reaction but could be the result of a trivial thermal decomposition of **1**. However, this is not the case. In fact, suitable control experiments showed no significant spectral change when **NCDs-βCyD@1** was stirred in the dark at 40 °C up to 1h (Figure S8).

Note that, in contrast with our recent work on **NCDs** with similar spectral features,^47^ no generation of ^1^O_2_ was observed under light excitation neither in the naked nor in the functionalized **NCDs**. This was proven by the absence of the typical NIR emission of ^1^O_2_ at 1270 nm. This result can be tentatively ascribed to a lower efficiency of population of the precursor excited triplet state in the present case.

### 3.3 Cell Studies

**NCDs-βCyD** and **NCDs-βCyD@1** exhibit red fluorescence upon λ_exc_ = 560 nm with low quantum yields (vide supra) but satisfactory to monitoring them in cancer cells in this unambiguous spectral region. Confocal fluorescence microscopy images were acquired after incubation of both nanoconstructs with Caco-2 colorectal adenocarcinoma cells. Figure 7A,B show clear cell internalization of both **NCDs-βCyD** and **NCDs-βCyD@1**. Co-localization experiments carried out with cells costained with either nuclear (Hoechst-33342) or mitochondrial (MitoTracker Green) targeting dyes demonstrate that both nanoconstructs do not internalize in the nuclei or mitochondria, but they remain confined mainly at the cytoplasmic level.

**Figure 7:**
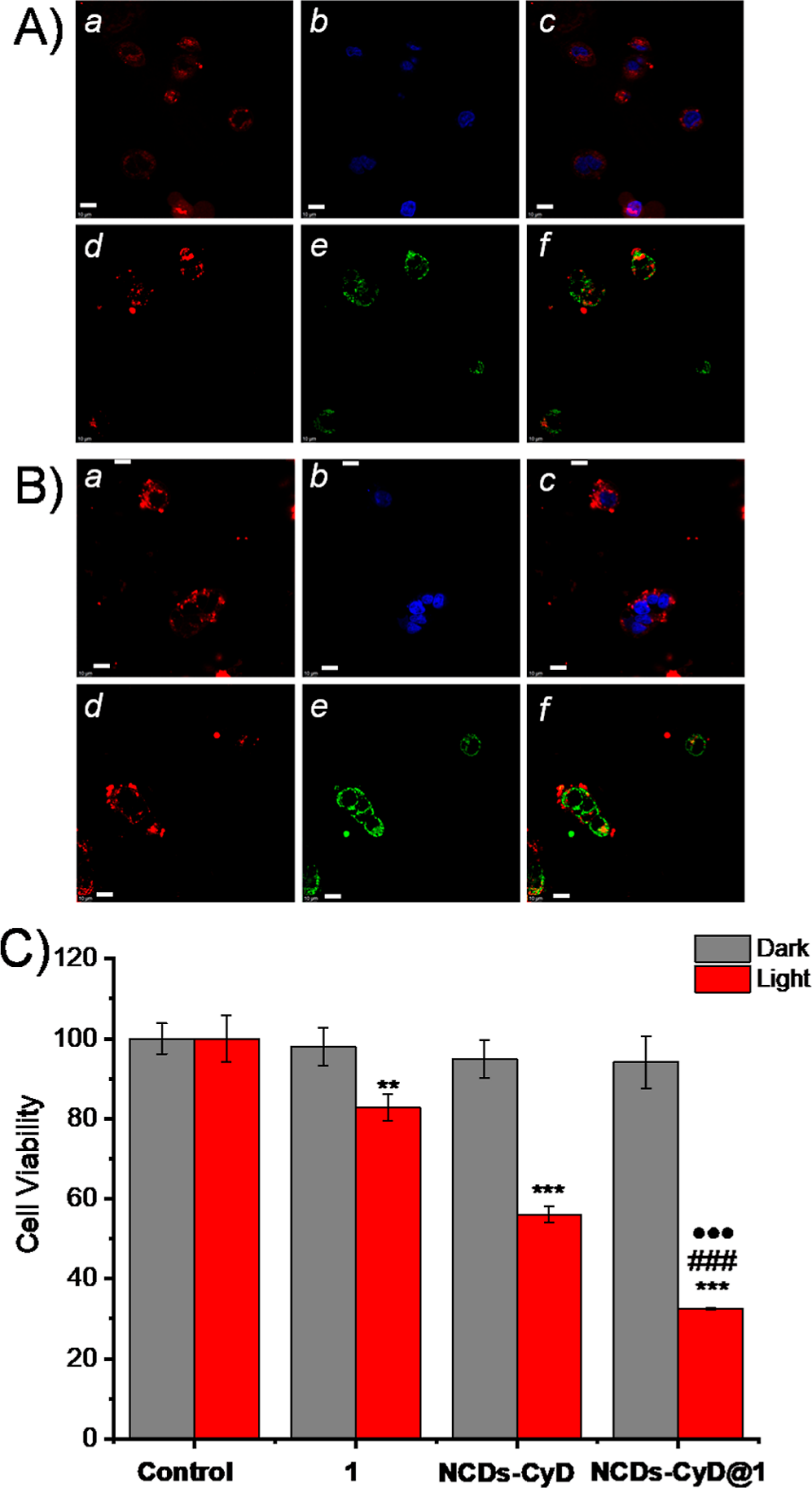
Confocal fluorescence microscopy images of Caco-2 cancer cells incubated with **NCDs-βCyD** (A) or **NCDs-βCyD@1** (B) and costained with NucBlue Dye Hoechst-33342 (panels **a**,**b**) and MitoTracker Green (panels **d**,**e**) for 2 h and acquired at: λ_exc_ = 561 nm, λ_em_ = 610–670 nm (red channel) (panels **a**,**d**); λ_exc_ = 405 nm, λ_em_ = 420–480 nm (blue channel) (panels **b**); λ_exc_ = 488 nm, λ_em_ = 500–520 nm (green channel) (panels **e**). Panels **c** and **f** show the overlay images. Scale bar = 10 μm. (C) Cell viability of the same cancer cells incubated for 2 h at 37 °C with **1**, **NCDs-βCyD** or **NCDs-βCyD@1** and either kept in the dark or irradiated with red light for 30 min. Statistical significance was defined as a *p*-value <0.05. Levels of significance were indicated as follows: *p* < 0.05 (*), *p* < 0.001 (**), and *p* < 0.0001 (***, ### or ●●●). [**NCDs-βCyD**] = 12.6 mg mL^–1^); [**1**] = 25 μM.

Experiments against the same cell lines evaluated the biological activity. The cancer cells were incubated with **NCDs-βCyD@1** and, for comparison, with **NCDs-βCyD** and the free **1**, and were either kept in the dark or irradiated with red light for 30 min. The results in Figure 7C show that all samples are well tolerated in the dark (cell viability > 90%), indicating good biocompatibility under these conditions. On the other hand, a considerable reduction of cell viability was observed for both **NCDs-βCyD** and **NCDs-βCyD@1** under red light illumination. Since both **NCDs-βCyD** and **NCDs-βCyD@1** samples are optically matched at the excitation wavelength (they absorb the same number of photons), and their photothermal efficiency was basically the same (see Figure 6A and related discussion), the higher level of photoinactivation induced by **NCDs-βCyD@1** can be reasonably the result of the involvement of a bimodal cell photokilling mechanism in neoplastic destruction, in which the NO photoreleased plays a role in addition/synergism to the photothermal action. In this regard, it should be noted that a slight but statistically significant reduction in cell viability was observed in the case of individual NOP **1** under irradiation (see Figure 7C). In view of the compound’s optical transparency to red light (see spectra **d**,**e** in Figure 4A), the origin of this effect remains unclear at the moment.

## 4 Conclusions

We have designed and prepared a red-light-absorbing nanoplatform based on βCyD-linked **NCDs**, *ca*. 3 nm in diameter, able to encapsulate, in a supramolecular fashion, a hydrophobic NOP that is otherwise activatable with blue light. The whole nanoconstruct is well dispersible in a water medium under physiological conditions and remains stable in a protein medium. The **NCDs** core serves as the sole red-light absorber, and its excitation in this highly biocompatible spectral range triggers NO release from the NOP guest, likely via intra-assembly photoinduced electron transfer, with the remarkable shift in the excitation wavelength of ca. 260 nm. The βCyD units integrated at the **NCDs** surface play a key 2-fold role: (i) ensuring no significant displacement of the NOP in a biological medium, and (ii) keeping the encapsulated NOP at appropriate distances from the NCDs, thereby avoiding undesired quenching effects that suppress its photoreactivity. Red light excitation of the supramolecular complex also results in a significant photothermal conversion, which is essentially the same as that observed in the absence of the NOP guest. Besides, the nanoconstruct exhibits satisfactory fluorescence emission in the unambiguous red region, enabling its tracking in cancer cells, where it localizes mainly at the cytoplasmic level. Cell viability experiments carried out under dark and light conditions demonstrated a clear bimodal mechanism of killing cancer cells with red light, as a result of the combined photothermal and NO photodynamic action.

Overall, the present nanoplatform provides one of the rare examples of CDs-based constructs that demonstrate the high potential of CDs not merely as inert delivery scaffolds but as effective light-harvesting antennas for photothermal gas therapy, inducing the simultaneous generation of heat and NO as transient cytotoxic species that do not suffer MDR issues under the exclusive control of the highly biocompatible and tissue-penetrating red light. In this view, the present nanoplatform is an ideal candidate for testing in a larger panel of cancer and healthy cells, and in vivo studies, which are underway in our laboratories.

## Supplementary Material



## Data Availability

All raw data are available, and the authors will submit them upon request.

## Notes

XPS analysis of **NCDs-βCyD** and binding energies and atomic percentages of C, N and O 1s for **NCDs** and **NCDs-βCyD**; Fluorescence emission spectra at different wavelength of **NCDs**; Fluorescence decay of **NCDs-βCyD@1;** Photolysis profile of **1** under blue light excitation; Absorption spectra of **1** in the presence of **NCDs** without and with BSA; Photolysis of **1** in the presence of **NCDs** under red light excitation; Thermal stability of **NCDs-βCyD@1** (PDF)
