# Supplementary material for: β‑Cyclodextrin-Coated N‑Doped Carbon Dots for Red-Light-Controlled Nitric Oxide Release and Photothermal Therapy
Source: ACS Appl Nano Mater. 2026 Jul 8;9(28):13577–88. doi: 10.1021/acsanm.6c02329 (PMC13436617; doi:10.1021/acsanm.6c02329)
Supplement: Supplementary file 1 [file an6c02329_si_001.pdf]

Supporting Information

β-Cyclodextrin-Coated N-Doped Carbon Dots for Red-Light-Controlled Nitric Oxide Release and Photothermal Therapy

Francesca Laneri,[†,¥] Cristina Parisi,[†,¥] Vittoria Andrigo,[‡] Gian Marco Leone,[#] Katia Mangano,[#] Ferdinando Nicoletti,[#] Marta M. Natile[‡] and Salvatore Sortino[†,*]

[†]*PhotoChemLab, Department of Drug and Health Sciences, University of Catania, I-95125 Catania, Italy.*

[‡]*ICMATE-CNR Institute of Condensed Matter Chemistry and Technologies for Energy, National Research Council, Department of Chemical Science, University of Padova, 35131 Padova*

[‡]*Department of Biomedical and Biotechnological Sciences, University of Catania, I-95125 Catania, Italy.*

*Email: ssortino@unict.it

[¥]These authors equally contributed to this work.

- **Figure S1……………………………………………………………………S2**
- **Figure S2……………………………………………………………………S3**
- **Figure S3……………………………………………………………………S3**
- **Figure S4……………………………………………………………………S4**
- **Figure S5……………………………………………………………………S4**
- **Figure S6……………………………………………………………………S5**
- **Figure S7……………………………………………………………………S5**
- **Figure S8……………………………………………………………………S6**
- **Table S1……………………………………………………………………S6**
- **Table S2……………………………………………………………………S6**

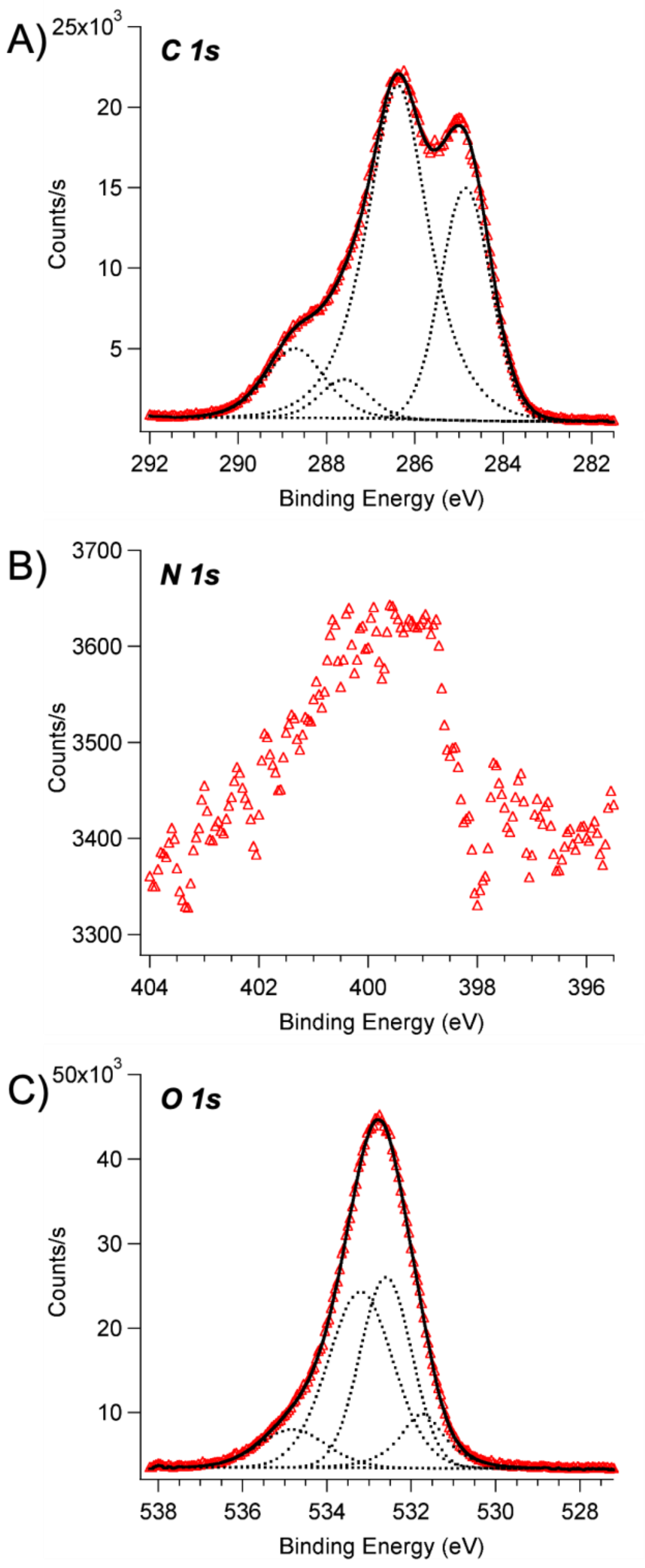

Figure S1. XPS analysis of **NCDs-βCyD**: high resolution spectra of (A) C 1s, (B) N 1s, and (C) O 1s. Red triangles for raw data; black continuum line and dashed lines for the fitting curves.

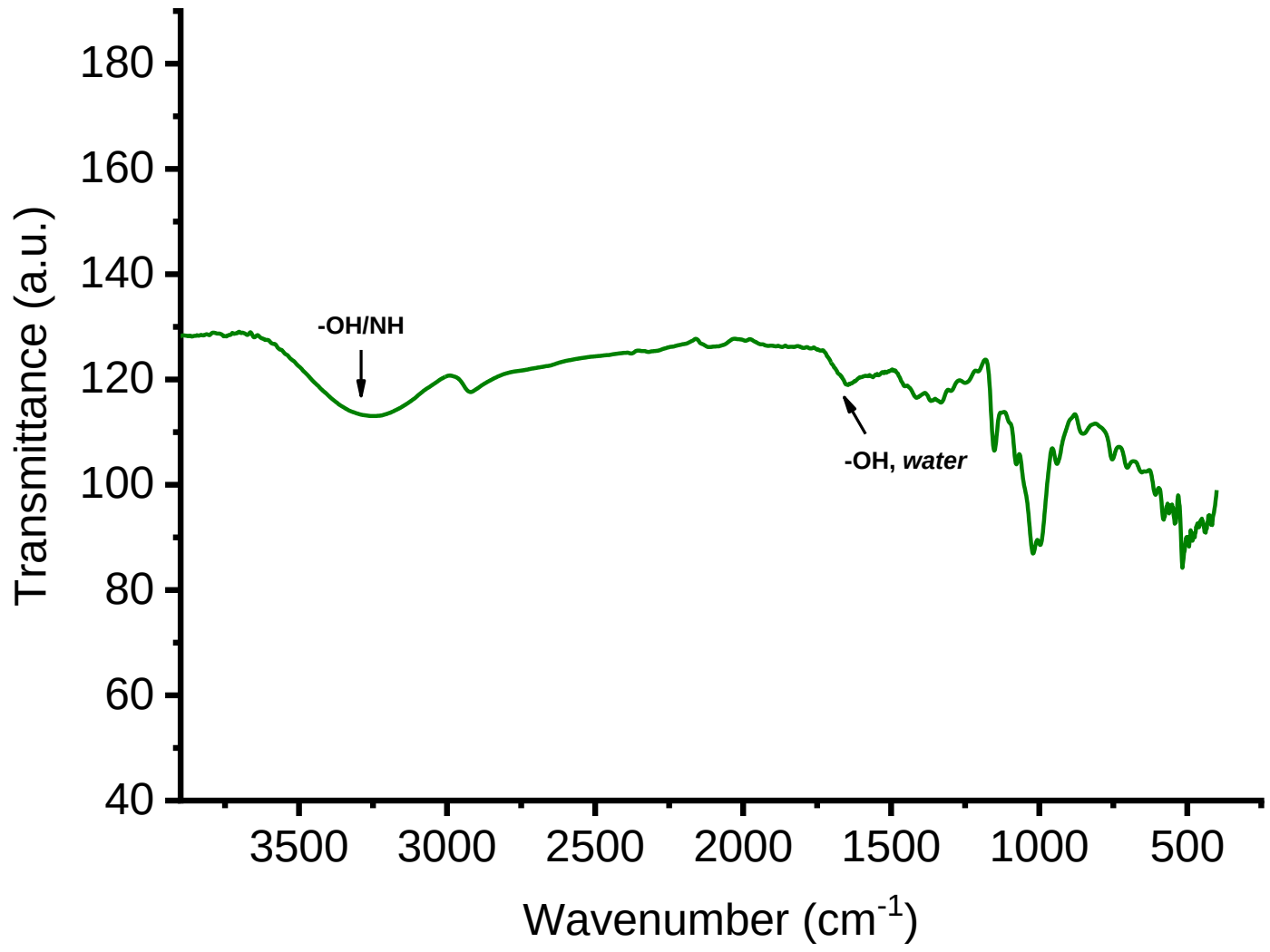

Figure S2. FTIR spectrum of a physical mixture of **NCDs** and βCyD.

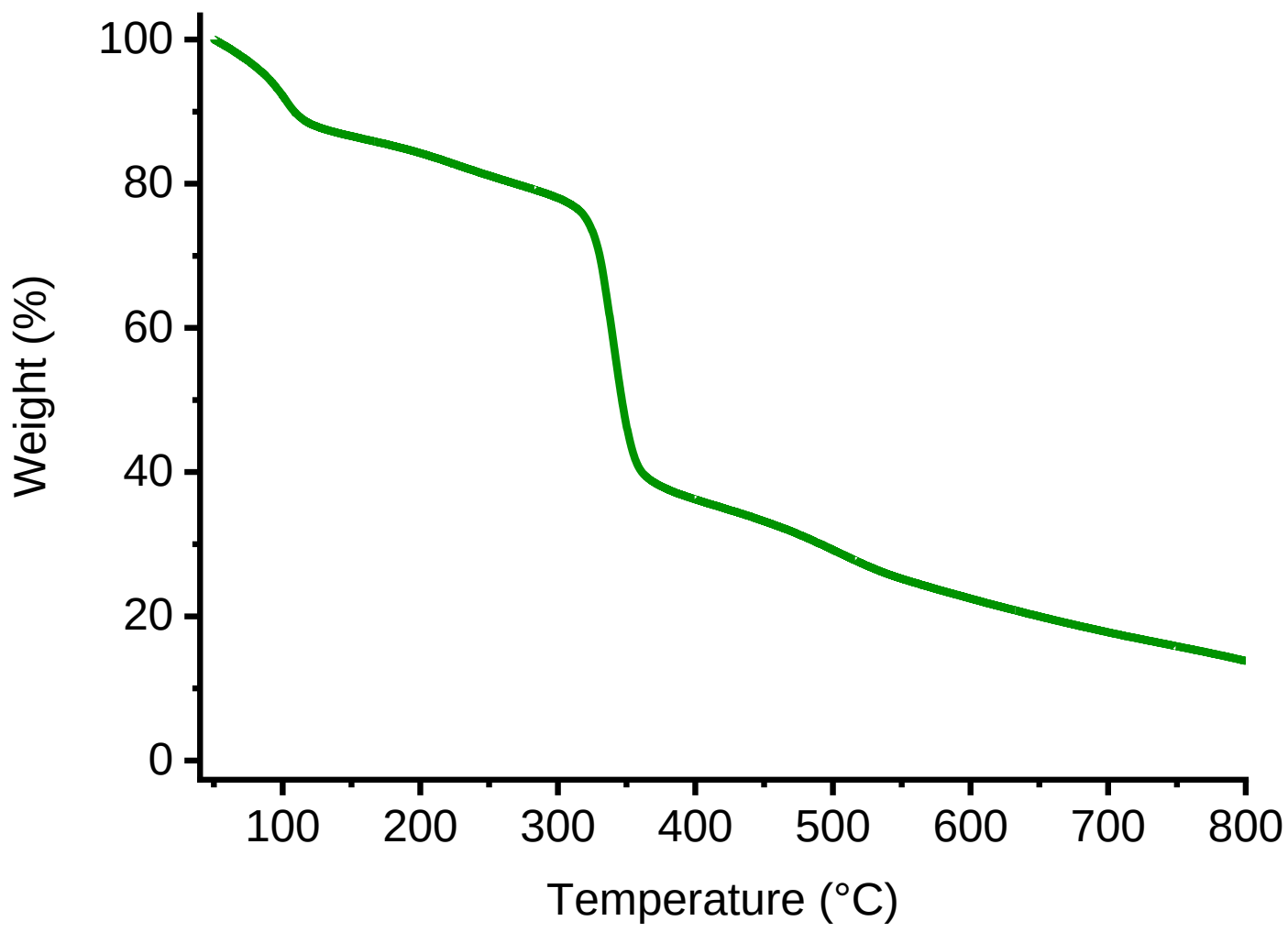

Figure S3. TGA analysis of a physical mixture of **NCDs** and βCyD.

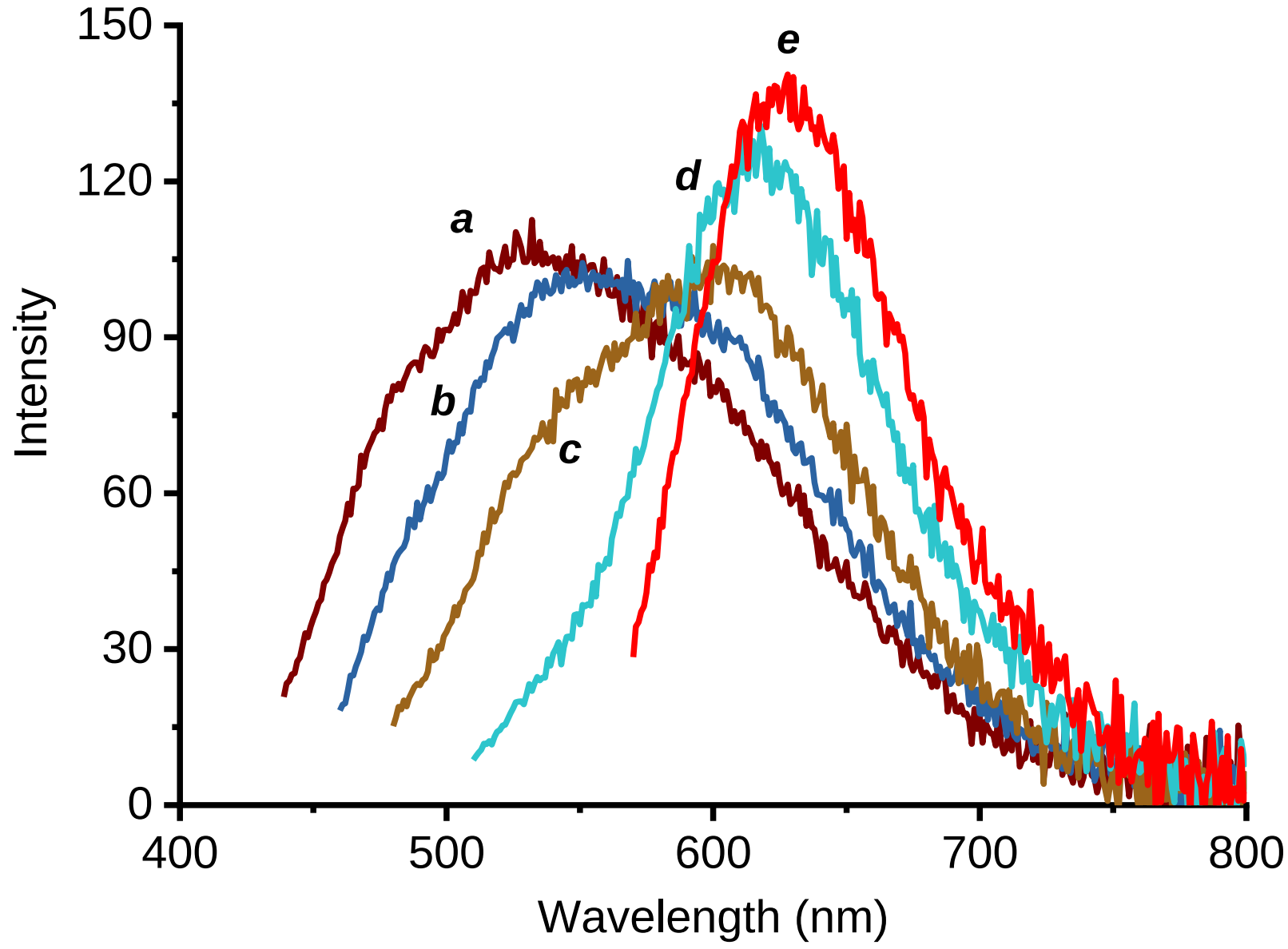

Figure S4. Representative fluorescence emission spectra of aqueous PBS (pH = 7.4, 10 mM) dispersions of **NCDs** at different excitation wavelengths: (***a***) = 430 nm; (***b***) = 450 nm; (***c***) = 470 nm; (***d***) = 500 nm; (***e***) = 560 nm. [**NCDs**] (0.7 mg mL^{-1}); T = 25 °C.

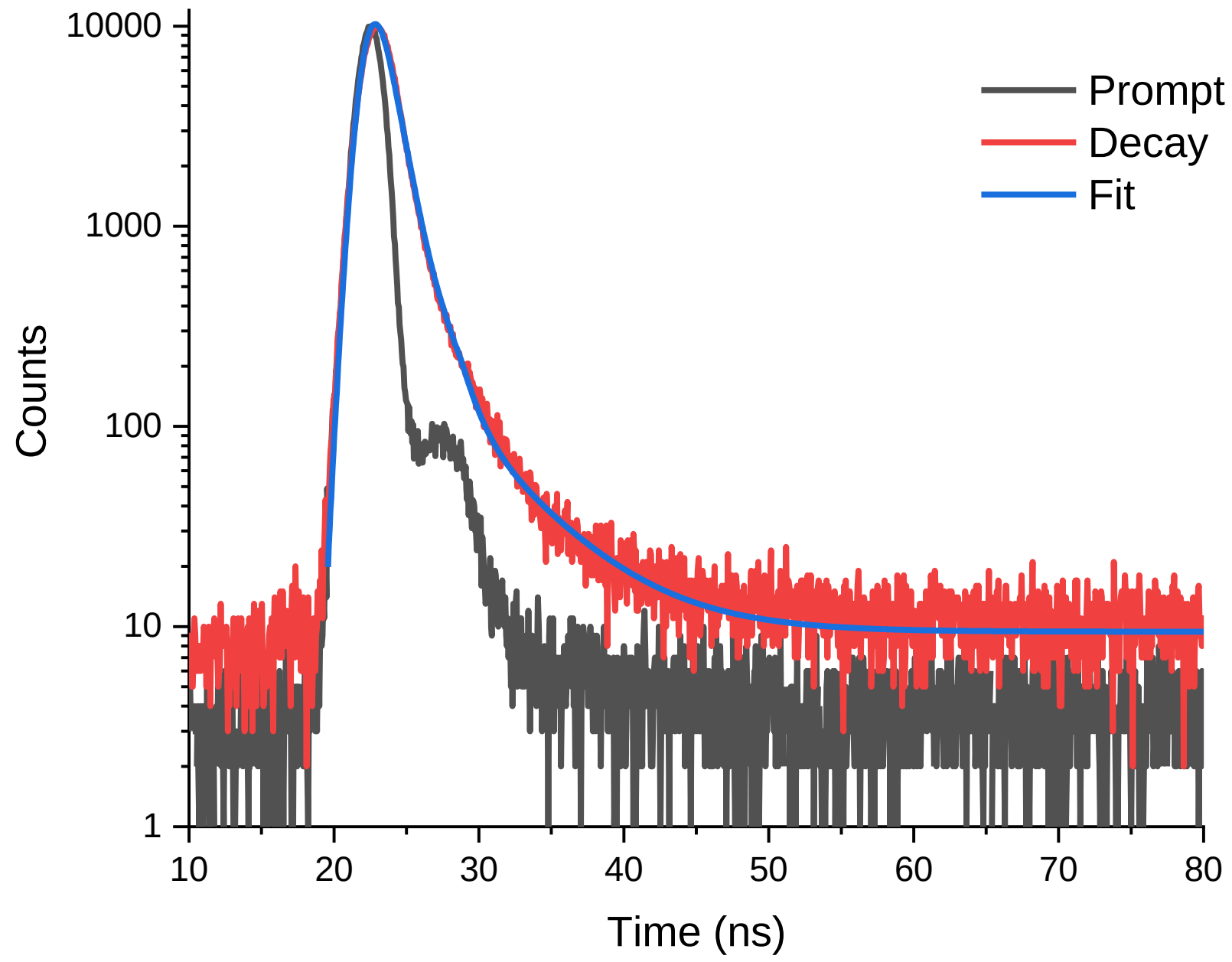

Figure S5. Fluorescence decay, λ_{exc} = 560 nm and λ_{em} = 630 nm, and the related bi-exponential fitting of an aqueous PBS (pH = 7.4, 10 mM) dispersions of **NCDs-βCyD@1**. [**NCDs-βCyD**] = 12.6 mg mL^{-1};); [**1**] = 25 μM; T = 25 °C.

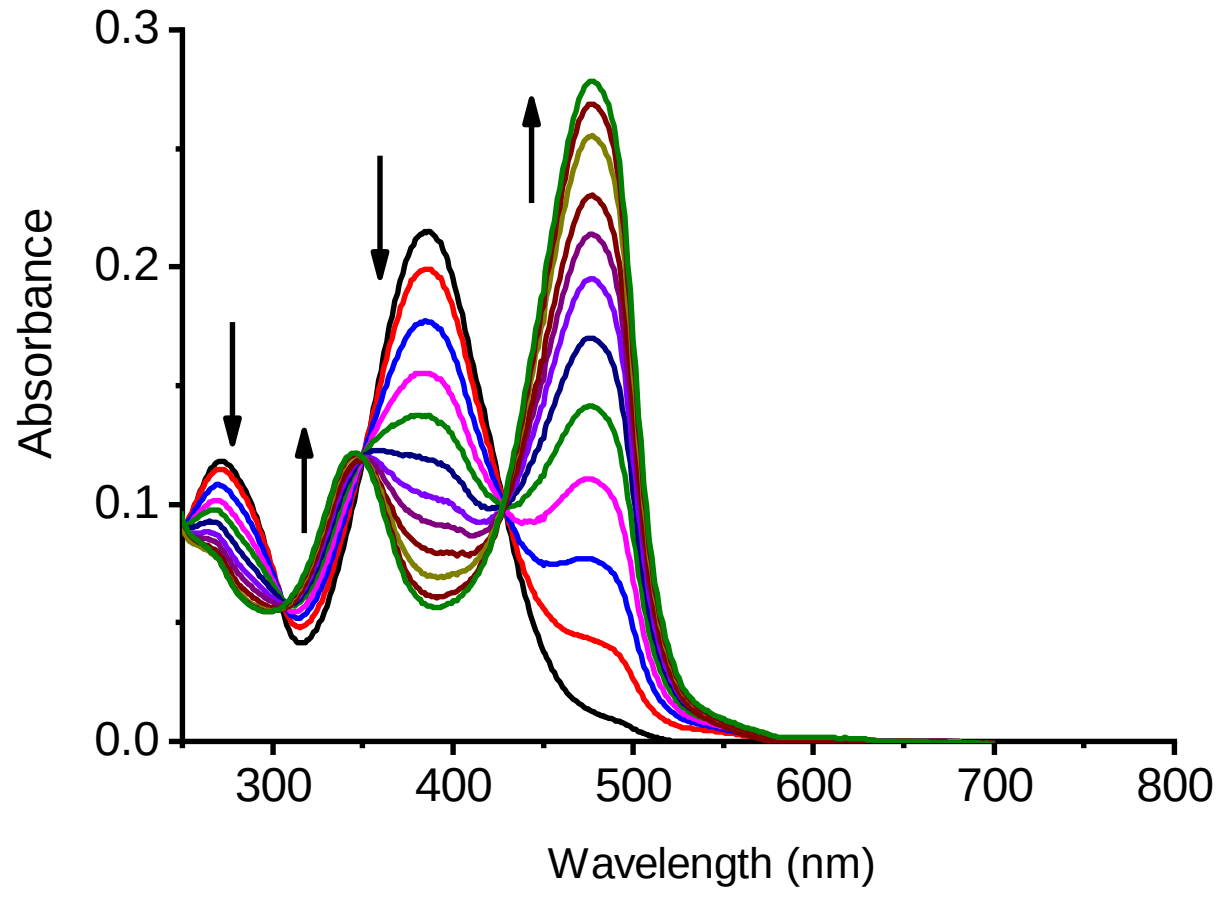

Figure S6. Absorption spectral changes observed upon exposure of a solution of **1** (15 µM) at λ_{exc} = 420 nm (*ca.* 20 mW cm^{-2}) for time intervals from 0 to 75 s. The arrows indicate the course of the spectral profile with the illumination time. H_2O: MeOH (1:1 v/v), T = 25 °C.

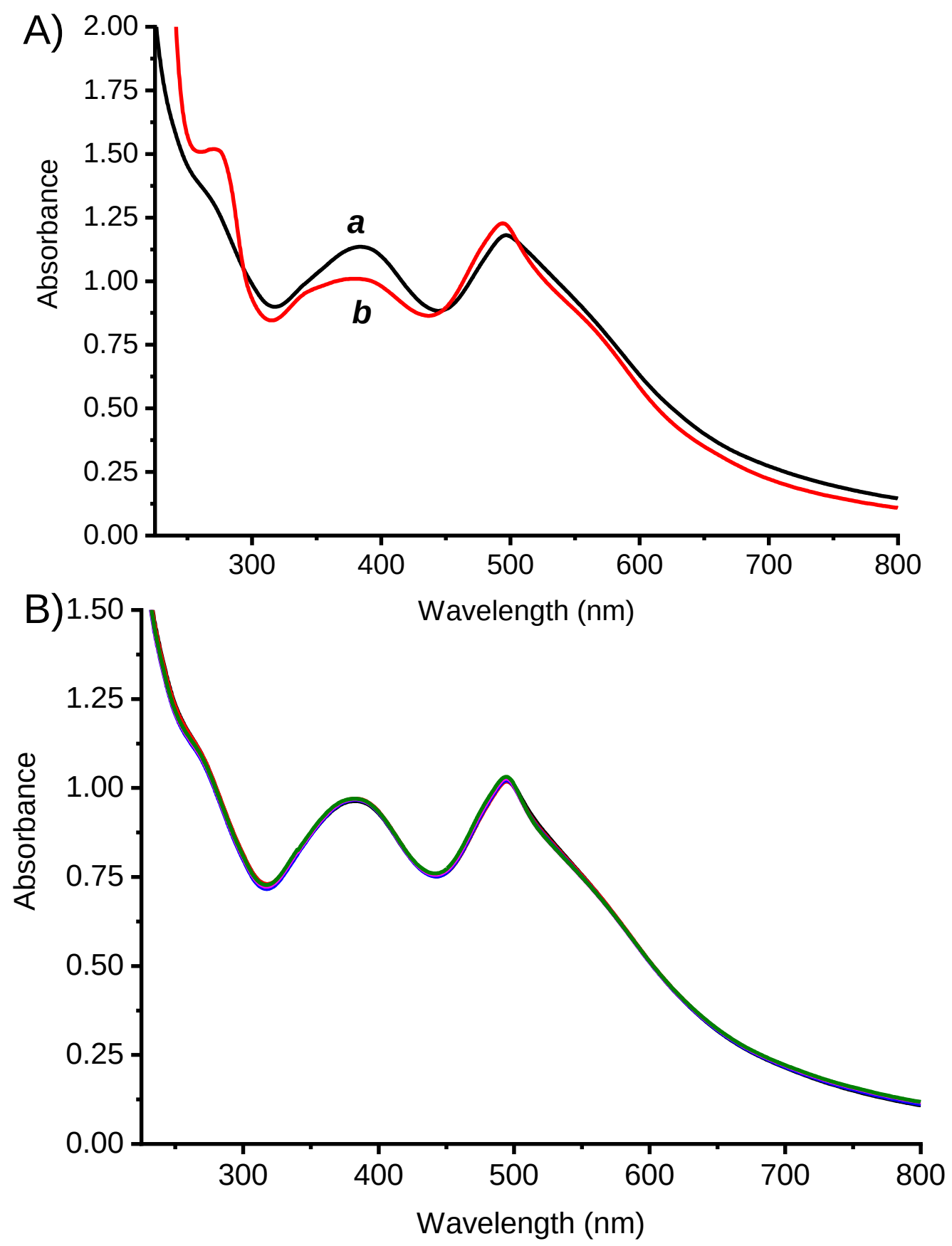

Figure S7. (A) Absorption spectra of a mixture of **NCDs** and **1** in PBS (pH 7.4, 10 mM) before (***a***) and after (***b***) 1 h of incubation with BSA at 37 °C. (B) Absorption spectral changes observed upon irradiation of an air-equilibrated PBS (pH 7.4, 10 mM) mixture of **NCDs** and **1** at λ_{exc} = 671 nm at different times from 0 to 90 min. [**NCDs**] = (0.7 mg mL^{-1}); [**1**] = 25 µM; [BSA] = 0.8 mg mL^{-1}. T = 25 °C.

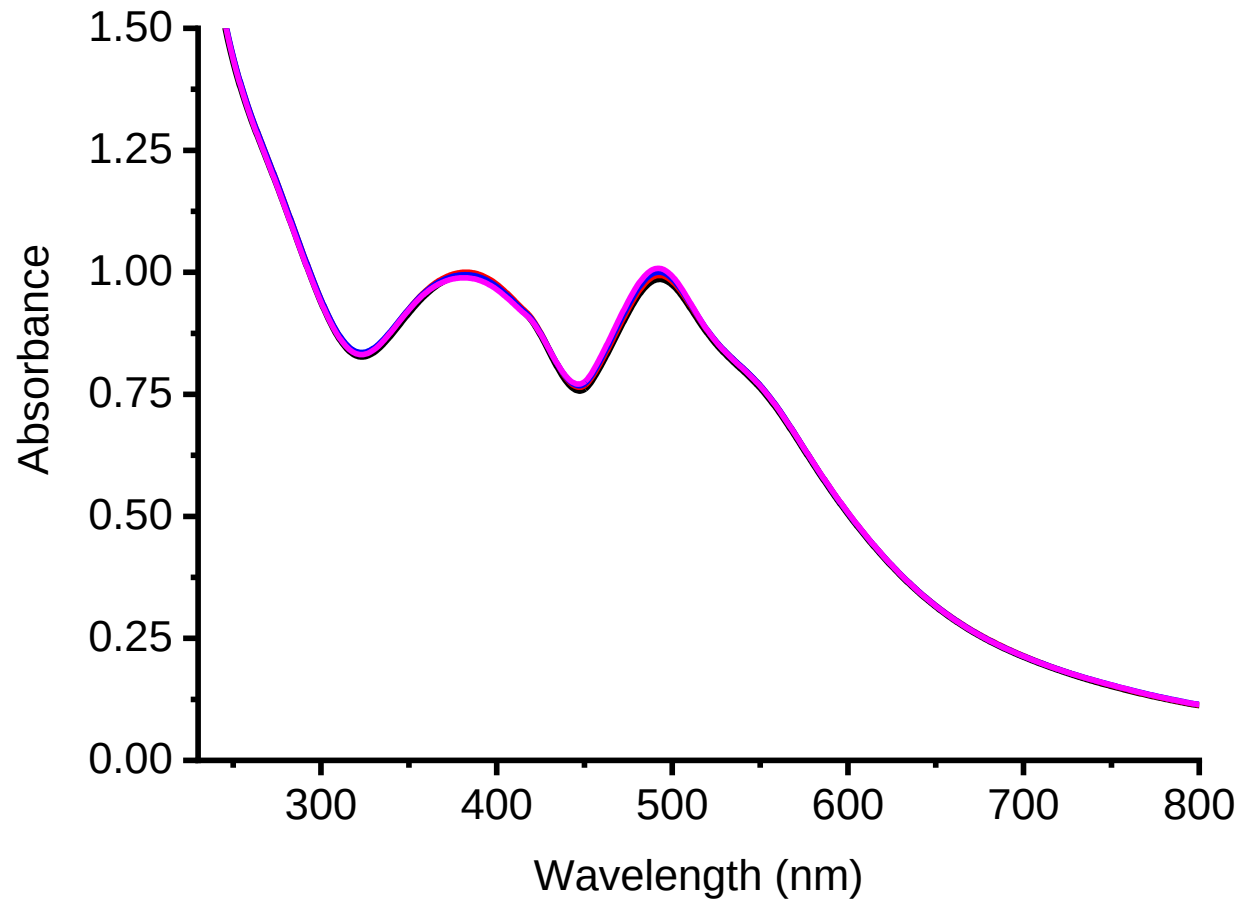

Figure S8. Absorption spectral changes observed at different times upon stirring of an air-equilibrated aqueous PBS (pH = 7.4, 10 mM) dispersion of **NCDs-βCyD@1** at 40 °C for 1h. [**NCDs-βCyD**] = 12.6 mg mL^{-1};); [**1**] = 25 μM.

Table S1. Summary of the fitting of C 1s, N 1s and O 1s core levels of pristine **NCDs**. Binding Energies (eV) of different species (eV) and corresponding atomic percentages are shown.

C 1s		N 1s		O 1s	
**BE (eV)**	**Atomic %**	**BE (eV)**	**Atomic %**	**BE (eV)**	**Atomic %**
284.8	31.2	398.4	0.6	530.8	1.8
285.8	23.8	399.4	1.7	531.8	9.4
287.1	6.5	400.1	2.7	533.2	11.5
288.8	10.5	401.5	0.3	--	--

Table S2. Summary of the fitting of C 1s, N 1s and O 1s core levels of **NCDs-βCyD**. Binding Energies (eV) of different species (eV) and corresponding atomic percentages are shown.

C 1s		N 1s		O 1s	
**BE (eV)**	**Atomic %**	**BE (eV)**	**Atomic %**	**BE (eV)**	**Atomic %**
284.8	19.1	398.9	0.5	531.5	3.7
286.4	37.4	--	--	532.4	13.3
287.6	6.9	--	--	533.0	15.9
288.7	3.2	--	--	--	--
